# Metabolism and Mouth-Disease

**Published:** 1910-11-15

**Authors:** Henry R. Harrower

**Affiliations:** Chicago


### METABOLISM AND MOUTH-DISEASE.

HENRY R. HARROWER, M.D., CHICAGO.

Since there is a very close relationship between the functions of the various organs of the body, it is not unreasonable to suppose that disorganization in one part will be accompanied or followed by disturbances elsewhere. From a study of conditions as they are to-day in both medicine and dentistry, however, it would seem that this important fact is not as fully appreciated as might be expected.

It is generally believed that certain more or less intractable diseases of the mouth or its contents are, to a greater or less degree, either accompanied by or associated with changes in the normal metabolic functions of the body. In spite of this comparatively few physicians are stimulated to look carefully into the mouth when evident errors of the metabolism are known to be present; nor do our dental confreres investigate the metabolism of those of their patients who are suffering from the all-to-common and intractable mouth-diseases.

This is not as it should be, for any local manifestation of disturbed function should serve as a reminder that a thorough investigation is not only necessary but imperative This is just as true of mouth diseases as of skin eruptions' joint difficulties or gastro-enteric disorders. All too often' Nature's glaring sign-posts are ignored.

Without a doubt there is a very close relationship between the condition of the buccal mucosa, the gums and teeth, as well as the tongue, and the blood which nourishes them. From time immemorial the condition of the tongue in disease has been used as an important diagnostic sign, and the study of this is given a prominent place in the text-books and current medical literature. For this reason I give it merely passing mention here.

It might be well here to quote a few lines from a valuable editorial entitled: "Gingivitis in Diabetes" printed in a recent issue of the *Journal*. "The importance of mouth symptoms in the acute infections, such as scarlet fever, diphtheria and measles, is recognized. It is less generally known, however, that in many constitutional conditions the mouth secretions and the mucous membranes covering the gums, cheeks, tongue, etc., furnish early and positive data for diagnosis."

"It becomes a matter of extreme importance, therefore, that the general practitioner shall examine the mouths of all patients, taking careful note of the mucous membrane of the cheeks, beneath the tongue, on the tongue itself, the roof of the mouth and especially the gums."

This subject is of paramount importance and one to which much more attention should be paid. Here we have a vital factor in the solution of a multitude of difficulties, not merely pyorrhea alone, nor, for that matter, mouth diseases *per se* but of a long list of common and uncommon conditions which embrace almost all the diseases known to medicine. It is a fact that as a profession we have not yet fully grasped the meaning of the Biblical statement, "The blood is the life." I do not mean by this that we do not appreciate the fact that life is dependent on the blood and its healthy condition, but the fact that in the majority of cases disease is due to toxic wastes which are present in the blood-stream and which unquestionably markedly lower its disease-resisting powers.

These poisons are not all of them sufficiently well known to have been isolated and named, but their frequent presence and easily demonstrable untoward effects are apparent to all that take the trouble to look for them.

Principal among these disease-producing toxins are certain acid substances which have been demonstrated to be closely related to indican and other products of intestinal putrefaction. Just what these substances are remains to be proved, but that they are acid in reaction and that their baneful influence is evident in the mouth as well as throughout the whole system is very quickly and easily proved.

Our methods of analyzing the blood and estimating its alkalinity are altogether too complicated for general use. In their place the examination of the urine has been found to be at once convenient, quick and easy; and the results obtained from such examinations will be found at times to be even startling. Probably the one factor obtained by the careful analysis of the urine which in most cases overtops its fellows is the acid index, and, strange to say, hardly more than a mention of it and the method of its estimation is to be found even in the most recent text books.

Let us for a moment consider the question of the urine analysis and the relations of some of the findings. The urine is usually acid in reaction and the average normal acidity ranges from thirty to forty degrees (each degree represents the amount of decinormal soda solution required exactly to neutralize 100 c.c. of urine). This acidity, we are told, is due to certain acid salts, principal among which is the acid phosphate of soda. We learn from the text books that the urinary acidity is very difficult to accurately estimate because of the trouble in securing an indicator which will be responsive to all the various acid salts, and probably for this reason the study of this most important finding has been largely passed over.

Phenolphthalein is the most satisfactory indicator and is used by the majority of investigators. The test is simplicity itself and is fully as accurate as many of the well-known and widely-used tests, as for example. the albumin tests of Esbach or Purdy, or the Doremus urea test. The acidity of the urine should always be determined from a portion of a complete twenty-four-hour specimen, and care should be taken to prevent, as far as possible, alkaline decomposition of the urine. Either a burette or my acidimeter[3] may be used. The latter is far more convenient for the physi-

3. Harrower, H. R.: New York Med. Jour., 1909, lxxxix, 1, 24.

cian or dentist in his office, while the former is probably better in routine laboratory work where large numbers of specimens are being handled.

In a paper[4] published last June I called attention to the frequency with which excessively acid urine accompanied indicanuria and a marked dimunition in the amount of urinary solids passed. A series of 250 analyses was mentioned in this paper. These findings, augmented by much further work along this line both by myself and several interested friends, was reported in a paper[5] read at the annual meeting of the Illinois State Medical Society held last month.

From my findings it would seem, to me at least, that we have conclusive evidence that in the study of the urinary acidity we have something of more than ordinary importance. The relation of this syndrome of findings—acidemia evidenced by a hyperacid urine, intestinal toxemia by indicanuria, and decreased metabolic activity by the frequent low urea index and general reduction in the total solids—to mouth-disease, is quickly found. It is safe to say that in a majority of patients suffering from pyorrhea are acidemics, and the most conclusive feature of this work is that therapeutic measures calculated to reduce the acidemia and eliminate the toxemia have a decidedly beneficial effect on the pyorrhea.

Many patients have been examined by me personally. Dr. Talbot has made hundreds of examinations and several others have by their work proved absolutely that there is decided and close connection between disturbed metabolism and mouth-disease.

I might go further and discuss the relations of the metabolism as evidenced by the urinary findings to diseases of the buccal mucosa, the pharynx and the tonsils, but this is a subject far too broad to be touched on as briefly as would be necessary here. I will close by relating a short but interesting case which was brought to my attention

4. Harrower, H. R. : Mec. Rec., New York, 1909, lxxv, 966.

5 Harrower, H. R. : The Clinical Significance and Relations of the Urinary Acidity, read at the 1910 meeting of the Illinois State Medical Society, Danville. Not yet published.

by Dr. A. H. Hoy. When he was visiting in Seattle last summer he was invited to see a little girl suffering from a membranous condition of the pharynx which seemed to be growing progressively worse in spite of heroic doses of diphtheria antitoxin, swabbings with silver nitrate and other antiseptics and the best treatment that one of the leading specialists could afford. Dr. Hoy asked if the urine had been examined. It had not. He then asked for permission to make an immediate examination of a specimen passed in the office. The acidimeter read 180 degrees. A twenty-four-hour specimen was saved, and the next afternoon was tested, showing practically the same degree of acidity.

To make a long story short, the child was treated with alkalies and within a few days the whole condition had cleared up and no evidence of the infection was to be seen save a marked hyperemia of the pharyngeal walls.

It is hoped that a wide-spread study of the relations between the disturbances of metabolism and mouth affections will shortly be inaugurated among the rank and file of the professions, and that the dentists as well as the doctors will come to see the important influence that the hyperacid state plays in disease causation in general and mouth disorders in particular.

### ABSTRACT OF DISCUSSION.

Dr. Eugene S. Talbot: I could recall many cases similar to that Dr. Harrower has reported in which by treating interstitial gingivitis, I have been able to clear up other conditions of the body from which the patients have suffered for many years. These patients are walking about yet they are ill. They are not ill so far as the general practitioner is concerned. He must find some lesion—some condition for which they must stay at home or in bed before taking treatment. There is no specialty of medicine wherein prophylaxis is more valuable than in dentistry. If we only knew and understood the mouth symptoms we could ward off disease in many instances. I have under way a series of experiments that are not completed, but I will give here a short report of them to show how these conditions—the urinary acidity and the indican will affect the tissues of the body.

For the past five years I have been making thes experiments on seven male patients over 45 years of age who have interstitial gingivitis to a marked degree. All are fleshy, weighing from 172 to 223 pounds. These patients all have painful micturition. They have been under my personal supervision all the time. All are suffering with acidemia and indicanuria. Four are high livers; three enjoy home life; five at times have urinary acidity of from 82 to 112 degrees; the other two do not exceed 60 degrees; all have abundant indican. These men are always about their business affairs. At times all suffering more or less with rheumatic neurotic pain, headaches, drowsiness, constipation, indigestion, gases in both stomach and bowels. These patients were experimented with in different ways. All were refused meat for a given time; all were refused acids and alcohols in all forms. Sodium bicarbonate was given in doses of from five to twenty grains three times a day one-half hour after meals until the acidity was reduced to below 35 degrees; from 30 to 40 degrees being normal. All patients were put on buttermilk. In four, the indican was reduced to a minimum and the pain subsided. In three indican remained in the urine. In these latter the buttermilk was withdrawn and intestinal antiseptics given. The indican was reduced by destroying the germs in the intestines. Meat was given for a period of a month or two as the case might be, and the urinalysis continued. Painful micturition returned. The experiments showed when the acidity was high and there was abundant indican, painful and frequent micturition occurred. After the urinary acidity was reduced, the pain continued; the reduction of indican in the urine, however, caused the pain to cease and the frequency to lessen. It was also found that the interstitial gingivitis could be more readily reduced and a permanency secured in not only all these patients but in others who were not under special study. It would seem that the decomposition of proteid substances in the intestines and bodily waste do not, when alone, cause the development of indican but by combination with intestinal germs which are not associated with nitrogenous substances to any extent, and which cannot be destroyed by the lactic acid of buttermilk. One thing is certain, indican and a high urinary acidity act as irritants in the blood stream, and the alveolar process and dental pulp, which are end-organs, are directly and quickly involved.

Dr. George V. I. Brown: It is evident, from the paper just read, that accompanying intestinal putrefaction and othe similar conditions, there is a general acidemia which affects the mucous membrane of the mouth in common with other parts. Buccal acidity may also be due to results of local fermentation. We have known for some time that dental caries is fostered in its incipiency by the formation of gelatinous plaques. No one, I believe, had satisfactorily shown just how they were formed until it occurred to Dr. Kirk to demonstrate that in the presence of lactic acid the mucin of the saliva is precipitated; he thus easily accounted for these plaques. In the same general way acidemia favors precipitation of mucin; it coates itself along the tongue and the mucous membrane of the cheeks, throat and other parts. Micro-organisms stick to it as flies stick to fly-paper. The rationale of tonsilitis, pharnygitis and other allied conditions is easily comprehended when one remembers that the tonsillar crypts are filled with this mucin and the micro-organisms thus protected are permitted to multiply under most favorable conditions.

Dr. Harrower: It has been proved conclusively that there is a close relation between prolapse of the abdominal viscera and indicanuria, and I would suggest that if Dr. Talbot would look into these three cases, he would find the patients "pot-bellied," and that if the abdominal contents were held in place the indicanuria would eventually disappear. He also referred to frequent and painful micturition. There is more to this than we think. This condition of hyperacidity or systemic acidemia is not only irritating to the kidneys but also to the urinary passages. I found in 25 per cent. of my first 250 cases that hyaline casts were present. Now hyaline casts are mucin, I believe; at all events, they are evidence of renal irritation, and it is not unreasonable to suppose that a condition which will cause the temporary presence of casts may be responsible for permanent kidney disorganization. I am not a crank on alveolitis, nor do I consider this condition of acidemia the sole cause of pyorrhea. Far from it. Unquestionably, local conditions play an important part and the presence of the acids of fermentation in the mouth have a greater bearing perhaps, than the general condition.—*Journal A. M. A.*, October 1, 1910.

